# Extracellular Matrix, a Hard Player in Angiogenesis

**DOI:** 10.3390/ijms17111822

**Published:** 2016-11-01

**Authors:** Maurizio Mongiat, Eva Andreuzzi, Giulia Tarticchio, Alice Paulitti

**Affiliations:** Experimental Oncology Division 2, Department of Translational Research, CRO-IRCCS, Aviano 33081, Italy; eandreuzzi@cro.it (E.A.); giuliatarticchio@libero.it (G.T.); apaulitti@cro.it (A.P.)

**Keywords:** extracellular matrix, angiogenesis, tumor microenvironment

## Abstract

The extracellular matrix (ECM) is a complex network of proteins, glycoproteins, proteoglycans, and polysaccharides. Through multiple interactions with each other and the cell surface receptors, not only the ECM determines the physical and mechanical properties of the tissues, but also profoundly influences cell behavior and many physiological and pathological processes. One of the functions that have been extensively explored is its impingement on angiogenesis. The strong impact of the ECM in this context is both direct and indirect by virtue of its ability to interact and/or store several growth factors and cytokines. The aim of this review is to provide some examples of the complex molecular mechanisms that are elicited by these molecules in promoting or weakening the angiogenic processes. The scenario is intricate, since matrix remodeling often generates fragments displaying opposite effects compared to those exerted by the whole molecules. Thus, the balance will tilt towards angiogenesis or angiostasis depending on the relative expression of pro- or anti-angiogenetic molecules/fragments composing the matrix of a given tissue. One of the vital aspects of this field of research is that, for its endogenous nature, the ECM can be viewed as a reservoir to draw from for the development of new more efficacious therapies to treat angiogenesis-dependent pathologies.

## 1. Introduction

The extracellular matrix (ECM) is composed by a variety of proteins, glycoproteins, proteoglycans, and polysaccharides that are endowed with distinct physical and biochemical properties [1]. For long the ECM has been viewed as a scaffold with mere mechanical properties aimed at maintaining tissue morphology. A growing amount of evidences support instead the notion that ECM is surprisingly versatile and dynamic and can profoundly affect cell behavior [2]. In fact, through direct or indirect mechanisms the ECM modulates the function of the adjacent cells during development but also in the adulthood under normal as well as pathological conditions [3]. For its crucial role in assuring proper tissue homeostasis and function, the deposition and remodeling of the ECM components are tightly regulated [4]. If this regulation is lost, the matrix loses its organization and this leads to an impaired cell behavior and to a consequent break down of tissue function and homeostasis. In fact, abnormal ECM is one of the most distinctive traits that can be observed in many pathologies, including fibrosis and cancer [5]. Cancer is one of the major causes of mortality accounting for millions of deaths worldwide. Several ECM components have been shown to play a critical role, not only during its onset but also during metastasis, which is responsible for the 90% of all the cancer-related deaths [6,7,8,9,10]. In this context, the activation of proteases leads to the release of matrix fragments that act directly on cancer cells influencing their viability, apoptotic rate and/or metastatic potential [11,12,13,14,15,16]. Matrix remodeling leads also to the release of growth factors and cytokines of which the ECM represent a vital reservoir. The thus altered tumor microenvironment not only directly affects tumor cell behavior, but also other important processes such as angiogenesis. Angiogenesis, the process of the formation of new blood vessel from pre-existing vasculature, plays an indispensable role both in physiological and pathological conditions. It occurs during development and maturation, as well as during the healing of injured tissues and reproduction in the female population and is indispensable to grant the supply of nutrients and oxygen to the tissues. The angiogenic process is also usurped by a number of pathologies including inflammation, autoimmune diseases and cancer [17]. Notably an imbalanced vascularization can contribute to the development of these diseases [18]. In autoimmune diseases an excessive angiogenesis can promote inflammatory responses and, as a consequence, exacerbate the disorder. In cancer, angiogenesis is required to allow and efficient growth of the restlessly proliferating tumor cells. The imbalanced angiogenic signals released during tumor onset and progression lead to the development of aberrantly tortuous and leaky vessels. The altered vasculature not only represents an important root for metastatic spreading, but also affects the delivery of chemotherapy drugs [19,20]. Along these lines, a recent view about the therapies targeting angiogenesis is that they should induce the formation of a proficient vasculature that would favor the distribution of the chemotherapy drugs within the tumors. Angiogenesis can be not only an accomplice of the disease, but also be its direct cause as demonstrated for macular degeneration and diabetic retinopathy [21].

For its primary role in tissue trophism and homeostasis, the angiogenic process is tightly regulated by a plethora of factors. In fact, the list of molecules affecting angiogenesis is growing and includes growth factors, bioactive lipids, complex polysaccharides and also several ECM components. The most important cell type activated during the angiogenic process is the Endothelial cell (EC). These cells form the inner cell lining of blood vessels and are present as a single-layered epithelium adjacent to the lumen of the vessels. ECs are normally quiescent and characterized by a very low proliferative rate. In part this is due to the strong anchorage of these cells, to the tight cell junctions that grant structural continuity and limit vascular leak. The release of growth factors and the onset of strong pro-angiogenic stimuli loosen these restrictions. As a consequence, ECs change their behavior and begin to proliferate and migrate invading the surrounding tissues. This is followed by a resolution state where the ECs re-acquire the quiescent state. Despite a gradient of cytokines or other agonists is necessary to prompt EC migration, the movement of these cells is strictly dependent on their adhesion to the ECM and also for this reason these constituents play a key role in this process [22,23,24]. The number of ECM molecules that have been shown to influence angiogenesis is increasing and this depends not only on their adhesive properties but also due to their intrinsic capacity to directly affect EC function. Given the variety of ECM constituents, to study the contribution of the microenvironment in this context is extremely complex. To exacerbate the scenario, the fragmentation of ECM molecules occurring during remodeling leads to the formation of peptides that can often display opposite effects compared to the intact molecules of origin [25].

**Figure 1:**
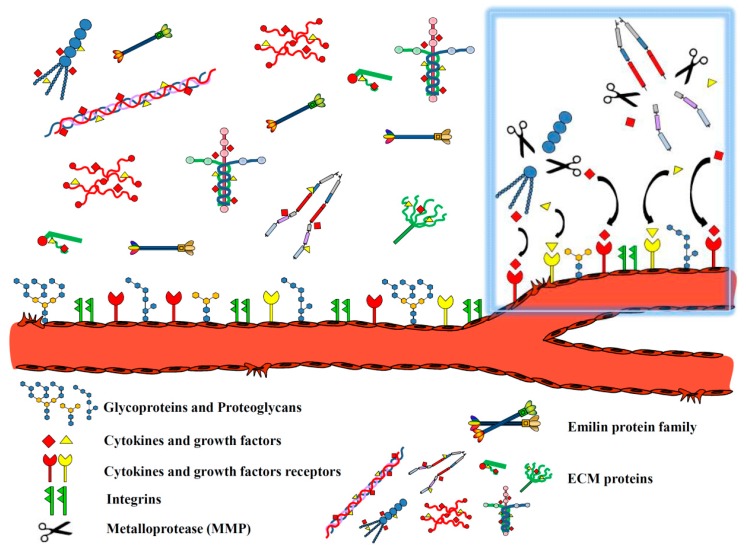
Schematic representation of key extracellular matrix (ECM) components involved in angiogenesis. On the left the major classes of ECM proteins that exert a role in angiogenesis are reported. The mechanism of growth factor release from ECM molecules through the action of proteases, activated upon angiogenic stimulus, is highlighted within the baby blue rectangle.

The aim of this article is to review a number of ECM molecules displaying a prominent role during angiogenesis and to summarize the major mechanisms impinging on EC function thus affecting the formation of new blood vessels. A schematic representation of the major classes of ECM molecules involved in angiogenesis is reported in Figure 1 and the molecules/fragments taken into account along with their respective functions summarized in Table A1.

## 2. Thrombospondins: Endogenous Angiogenesis Inhibitors

Thrombospondins (TSPs) are a family of large ECM glycoproteins including five members (TSP-1 to TSP-5) [26]. TSP-1 and TSP-2 have been extensively studied for their antiangiogenic properties. TSP-1 was the first to be identified as a naturally occurring angiogenic inhibitor [27]. Since this seminal paper, numerous have been the studies indicating that both TSP-1 and TSP-2 inhibit angiogenesis in multiple in vitro and in vivo assays [28,29]. Accordingly, the over-expression of these two molecules by tumor cells is associated with an impaired tumor growth in mice [30,31,32,33]. Interestingly, the simultaneous over-expression of both the molecules leads to additive effects, suggesting that TSP-1 and TSP-2 act by distinct mechanisms [31]. TSP-1 and TSP-2 specifically induce apoptosis in microvascular ECs leading to an impaired EC function and prejudiced tubule formation in vitro [34,35]. The molecular mechanism involves the binding to the transmembrane glycoprotein CD36 [34,36,37]. The interaction with the receptor results in the association of Src family kinases fyn or yes, which in turn lead to apoptosis through the phosphorylation of c-jun N-terminal kinase (JNK) and caspases [38]. It is interesting to note that the ECM non only supports cell survival through integrins engagement, but can also negatively affect cell viability in some circumstances.

In order to pinpoint the region(s) of TSP-1 responsible for the anti-angiogenic activity, synthetic peptides based on the protein sequence have been created and employed [28,35,39]. One of the first synthetic peptide generated contains the CSVTCG amino acidic sequence and was active in inhibiting the FGF-2-induced neovascularization of the rat cornea [28]. Some of the active sequences mapped are common to both TSP-1 and TSP-2, which indicates that they share common mechanisms of action. TSPs determine the EC phenotype through the binding to cell membrane or cell-associated molecules including integrins, CD36, CD47 and proteoglycans [40]. The multiple interactions on the EC surface may result in the assembly of molecular complexes able to trigger several signal transduction pathways, which may be the key of their strong effects. TSP-1 interacts and modulates the function of several cytokines/growth factors, proteases and ECM molecules [41], which suggests the possibility to develop new anti-angiogenic drugs [39,42,43]. Both TSP-1 and TSP-2 contain the thrombospondin type 1 repeat (TSR), an essential module for the binding to β_1_ integrins, to CD36 and to the transforming growth factor β (TGF-β) [44]. Notably, a therapeutic agent based on the second TSR has been developed [44] and is in clinical trials for several malignancies [45,46].

Thus, the studies on TSP-1 and TSP-2 have lead to important findings that have the translational potential to generate new promising tools for clinical practice. The drugs based on endogenous antiangiogenic molecules may in fact enclose the advantage not to induce harsh side effects during the treatments.

## 3. Fibronectin: Key Function in Pathological Angiogenesis

Fibronectin is strongly expressed around developing vessels during embryogenesis but is barely detectable in the adult vasculature [47]. Nonetheless, during pathological angiogenesis, the expression of the molecule is turned back on [48,49]. Fibronectin forms dimers composed of two similar monomers, which are organized into type I, type II and type III repeats. Different isoforms are present and are generated by alternative splicing within the EIIIA, EIIIB and V regions.

Fibronectin interacts with different ECM components including tenascin, thrombospondin, heparin, collagen and fibrinogen [50]. However its effects do not only rely on the molecular network with which fibronectin is connected. In fact, through the RGD motif, fibronectin binds to integrin receptors and in particular to integrin α_5_β_1_, which is markedly up-regulated during tumor-associated angiogenesis [51]. The deposition of fibronectin in a 3D cell-derived ECM appears to be imperative for matrix assembly and vascular morphogenesis [52]. Fibronectin prompts EC survival, whereas the blockage of its polymerization induces an impairment of EC proliferation and tube formation both in vitro and in vivo [48,53]. The importance of this molecule in angiogenesis is highlighted by the fact that, whereas the lack of the splice variants EIIIA or EIIIB does not lead to obvious defects, the double knockout mice show hemorrhages and severe vascular defects [54,55,56].

Given its wide distribution and interaction with other molecules involved in angiogenesis, it is likely that the altered expression of fibronectin observed in a number of pathologies such as fibrosis and cancer, may significantly impact on the development of new vessels and on the efficacy of anti-angiogenic therapy.

## 4. Collagens: A Major Source of Anti-Angiogenic Fragments

The synthesis and deposition of various collagens is known to affect EC survival and vessel formation. Those principally involved in angiogenesis are: type I, type IV, type XV and type XVIII collagen.

Type I collagen is the main ECM constituent to which proliferating ECs are exposed in an injured tissue. The interaction of collagen I with α_1_β_1_, α_2_β_1_, α_ν_β_3_ and α_ν_β_5_ integrins induces the activation of Mitogen-Activated Protein (MAP) kinase pathway supporting EC survival [57,58]. Moreover, the binding of collagen I to α_1_β_1_ and α_2_β_1_ integrins, suppresses c Adenosine Monophosphate (cAMP)-dependent Protein Kinase A (PKA), resulting in the reorganization of the actin fibers and in cell shape changes [59]. Collagen I is also important for lumen formation since it is required for the coalescence of pinocytic intracellular vacuoles [60,61]. Thus, a major role of this collagen can be recognized in the first stages of the angiogenic process.

Type IV collagen is one of the major constituents of the basement membrane [62,63], and is composed of six genetically distinct α chains (α 1–6) [64]. EC adhesion and migration are prompted by the triple-helical part of collagen IV, whereas the non collagenous domain NC1 does not support EC migration. Instead, the NC1 domains compete with intact collagen IV for the binding to integrins, inhibiting EC proliferation and tube formation [65,66]. In the early stages of tumor development, collagen IV exerts a pro-angiogenic function, once cryptic domains are exposed by MMP cleavage [67]. In contrast, in the late stages, it displays anti-angiogenic properties due to the release of arresten, canstatin and tumstatin derived from the α1, α2 and α3 chains, respectively [68,69,70]. Arresten inhibits EC migration and tumor growth through the engagement of integrin α_1_β_1_ and inhibition of MAP kinase [65,71]. Similarly, canstatin also inhibits EC migration and tube formation [72] and impairs angiopoietin-1-induced angiogenesis and lymphangiogenesis [73]. The mechanisms of action involve the binding to integrins α_ν_β_3_ and α_ν_β_5_, and inhibition of Protein Kinase B (PKB) also known as Akt focal adhesion kinase (FAK) and mammalian Target of Rapamycin (mTOR) [74]. In addition, canstatin induces Fas-dependent EC death, similarly to what observed with TSPs, despite through different mechanisms [75]. Finally, tumstatin binds to α_ν_β_3_ integrin though two distinct sites and displays anti-angiogenic and anti-tumor activity [76,77]. Thus, as observed for other ECM molecules, collagen IV displays an opposite function once the angiogenic stimulus is turned on and the proteases are activated. This likely represents a feedback mechanism able to halt the persistence of the pro-angiogenic signals. It can also be pointed out that the various fragments activate different molecular pathways thus amplifying the mechanisms of action.

Type XV collagen is a component of the vascular basement membrane [78]. Its carboxy-terminal globular Non-Collagenous (NC) domain called restin displays a high homology with endostatin and inhibits EC migration [79,80,81]. Type XV collagen deficient mice develop normally but display collapsed capillaries and EC degeneration in the heart and skeletal muscle, and this suggests that the molecule could exert an important role in microvessels stabilization [82].

Type XVIII collagen is found in different epithelial and vascular basement membranes. The proteolitic cleavage of the NC1 domain of the α1 chain by cathepsin L, B and K or MMPs releases the anti-angiogenic fragment endostatin [83,84]. Endostatin significantly reduces EC invasion in vitro through the blockage of matrix metalloproteinase 2 (MMP-2) [85]. The fragment also inhibits FGF-2 and VEGF-induced EC proliferation and migration [86,87]. Relying on its anti-angiogenic properties, endostatin displays potent anti-tumoral effects [88,89,90,91]. Remarkably, the treatment with the recombinant fragment induces vessel normalization and, as a consequence an improved chemotherapy efficacy [92]. Clinical trials have also been attempted with seesawing results: some deluded the expectations [93,94], whereas others demonstrated a potential benefit [91,95]. It is possible that this may depend on the tumor type and the quality of the vasculature characterizing each tumor. In addition, the microenvironment of the specific tumors and its intrinsic matrix composition may also impinge of the activity of endostatin.

Taken together, first the importance of this family of molecules in angiogenesis is highlighted by the fact that some of the members are intrinsic components of the vascular basement membrane. Given that vessel integrity affects vascular efficacy, an altered expression of these molecules may profoundly affect drug delivery and efficacy. Second, the pivotal role of this family in this context is that it represents an important source of fragments displaying strong angiogenic activities. As discussed for TSPs, these fragments represent a vital reservoir for the development of new drugs that may display high anti-angiogenic activity and low toxicity.

## 5. Laminins: Multiple Chains for Multiple Functions

The expression of laminins varies between cell types and during development [96]. Several sequences within the α, β and γ chains of laminins play a role in angiogenesis. The activation of MMP-9 following hypoxia leads to the degradation of laminin, which associates with an enhanced vascular pruning [97]. The RGD and IKVAV peptides present within the laminin sequence display important angiogenic functions, but to exert their activity they need to be unmasked by proteases [98]. The α4 (in particular laminin 411, α4:β1:γ1 and laminin 421, α4:β1:γ1), and α5 chains of laminin are the most abundant in the vascular basement membranes and thus play a major role in this context [99,100].

In particular, the α4 chain of laminin represents a high affinity ligand for α_ν_β_3_, α_3_β_1_ and α_6_β_1_ integrins and is required for EC adhesion and migration [101]. Interestingly, the α4 chain and its receptor Melanoma Cell Adhesion Molecule (MCAM) are highly expressed in the blood vessels of renal cell carcinomas and the levels of expression may serve to predict the patients’ outcome [102]. The importance of laminin 411 is also highlighted by the fact that, by activating the Notch-δ-like 4 signaling pathway, may regulate tip cell formation and affect vascular density [103]. On the other hand, laminin α5 chain-based synthetic peptides inhibit angiogenesis and block cell binding to FGF-2 [104]. Other studies report a complementary function for laminin α4 and α5: laminin α4 knockout mice show bleeding during embryogenesis resulting in a perinatal anemia, which is rescued 3 to 4 weeks postnatally once laminin α5 expression is turned on [105]. It has also been suggested that laminin 511 is required to maintain the β_1_ integrin-dependent anchorage of ECs in shear stress conditions and thus may be important to the formation of functional vessels [106,107]. In 3D EC cultures laminin is required for EC aggregation in end-to-end networks and the effect is α_6_ integrin dependent; the engagement of α_6_ integrin by laminin increases Vascular Endothelial Growth Factor Receptor 2 (VEGFR2) expression and Vascular Endothelial Growth Factor (VEGF) uptake by EC [108].

Laminin exerts an important role also during EC sprouting, in fact it has been recently demonstrated that, once bound to integrin α_6_β_1_, the receptor is sequestered from the podosome rosettes, key structures in sprouting angiogenesis [109].

Also for this family of molecules, characterized by different chain arrangements, the effect on angiogenesis is complex and occurs through the interaction and/or activation of major signaling pathways in ECs. As suggested by the results obtained with the use of the knockout models, the different molecules may display overlapping functions. Interestingly, since these molecules affect EC tip formation and sprouting angiogenesis they may be required in the early stages of angiogenesis. On the other side, the presence of these molecules in the blood vessels’ basement membrane suggests a role in the maintenance of vascular homeostasis.

## 6. Proteoglycans: Complex Functions from the Protein Core and Carbohydrate Chains

Proteoglycans (PGs) are complex macromolecules composed of a core protein carrying one or more covalently linked glycosaminoglycan (GAG) chains, whose number and type may differ to a great extent [110,111]. PGs perform multiple functions in cancer and angiogenesis by virtue of their polyhedric nature and their ability to interact with different ligands and receptors [112,113,114]. During tumor development and growth, PG expression is markedly modified within the tumor microenvironment impacting on cancer cell signaling and angiogenesis [115]. Heparan sulfate PG (HSPG) such as syndecans and glypicans are regulators of cancer progression and angiogenesis and serve as biomarkers for early detection and/or as pharmacological targets [116,117,118]. Notably, high concentrations of condroitin sulfate PG (CSPG), in particular versican and decorin, have been reported in the tumor stroma [111,119,120]. The major PGs affecting tumor angiogenesis are described below.

Perlecan is a ubiquitous multimodular proteoglycan consisting of five domains sharing homology with growth factors, immunoglobulins and adhesion molecules [121,122,123]. Given its widespread distribution [124,125] and its ability to interact with various molecules perlecan regulates various biological processes. Either via the protein core or the heparan sulfate chains, perlecan binds other ECM components as laminin, fibronectin and ECM1 and several growth factors including Fibroblast Growth Factor 1 (FGF-1), FGF-2, FGF-7, FGF-9 and FGF-18, FGF-binding protein, Platelet-Derived Growth Factor (PDGF), Hepatocyte Growth Factor (HGF), activin A, VEGF and progranulin [126,127,128,129,130,131,132]. The central role of perlecan in angiogenesis has been demonstrated by several independent studies and in various in vitro and in vivo models [133,134,135]. Collectively, these studies indicate that perlecan exerts a pro-angiogenic function by binding and presenting VEGF-A, PDGF and various FGFs to their cognate receptors and modulating their activity [136,137,138,139,140,141,142]. During tumor angiogenesis and cancer growth the perlecan protein core is degraded by proteases and the heparan sulfate chains are removed by heparanases. As a consequence, a plethora of growth factors trapped within the molecule are released affecting angiogenesis and cancer progression [114,143]. Interestingly, perlecan expression is often deregulated during cancer progression, generally leading to enhanced tumor invasiveness [144,145,146,147]. This may likely depend both on its structural role in the vessels’ basements membranes and its direct role in affecting angiogenesis.

Endorepellin is the C-terminal processed form of perlecan and, in contrast to the pro-angiogenic N-terminal domain, displays an opposite function: it inhibits EC migration, capillary morphogenesis, and in vivo angiogenesis [148,149,150,151]. The angiostatic function of endorepellin is in part due to its interaction with α_2_β_1_ integrin and activation of a signaling cascade leading to dissolution of the actin cytoskeleton, disruption of focal adhesions and the inhibition of EC migration [149]. Furthermore, the engagement of α_2_β_1_ integrin by endorepellin triggers the activation of the tyrosine phosphatase Src Homology region 2 domain-containing Phosphatase-1 (SHP-1) which, in turn, dephosphorylates and inactivates various Receptor Tyrosine Kinases (RTKs) including VEGFR2 [152]. Endorepellin is also able to directly interact with VEGFR2 at the EC surface inducing the transcriptional repression of HIF-1α and VEGF-A [153]. The binding of endorepellin with VEGFR2 is concurrent with its binding to α_2_β_1_ integrin and the dual receptor antagonism concur in the inhibition of EC migration and blood vessel maturation [154,155]. More recently, it was also demonstrated that endorepellin evokes autophagy via VEGFR2 signaling contributing to the angiostatic function of the fragment [156,157]. Thus, similarly to what observed with collagens, the cleavage of perlecan gives rise to a fragment displaying an opposite function, which may serve to counterbalance the angiogenic stimulus.

Decorin is a member of the small leucine-rich proteoglycan (SLRP) family and its structural features enable the binding to numerous other ECM molecules, growth factors and cytokines modulating angiogenesis with different mechanisms [158]. The binding to type I, type VI collagen and fibronectin affects collagen fibril formation and modulates the ECM rigidity and stiffness, structural properties known to influence angiogenesis [159,160,161]. As other ECM molecule, decorin modulates the activity of pro-angiogenic growth factors including VEGF, PDGF, FGF, Insulin Growth Factor (IGF) and angiopoietin by sequestering them and influencing their availability [162,163,164,165]. Decorin controls also the bioavailability of TGF-β whose release depends on the activity of different proteases involved in angiogenesis [166,167]. Furthermore, soluble decorin is a high affinity antagonistic ligand for several key receptors tyrosine kinases including Epithelial Growth Factor Receptor (EGFR), HGF Receptor, IGF-1 Receptor and VEGFR2 [168]. The engagement of decorin with cell surface receptors can either activate or inhibit the function of the receptor, depending on the physiological state of the tissue [169]. More recently, decorin has emerged as a soluble pro-autophagic cue [170]. This effect is mediated via a direct interaction with VEGFR2 which causes activation of AMP kinase signaling that ultimately culminate in a Paternally-expressed gene 3 (Peg3)/Beclin1/microtubule-associated proteins 1 Light Chain 3A (LC3) dependent autophagic program [171,172]. Thus, depending on the microenvironment in which angiogenesis occurs, decorin can exhibit either a pro- or an anti-angiogenic activity [165]. Nevertheless, in tumor-associated angiogenesis and in various inflammatory processes, the antiangiogenic activity is predominant, providing a potential basis for the development of decorin-based therapies [173,174,175].

Biglycan shares about 65% overall homology with decorin. Through the binding to VEGFA and activation of VEGFR2 biglycan exerts a pro-angiogenic stimulus in fracture healing processes [176]. The pro-angiogenic function is also the result of its interaction with and inactivation of endostatin [177]. In colon cancer, the over-expression of biglycan induces VEGF up-regulation and angiogenesis and thus the molecule may be a promising target for the development of anti-angiogenic strategies [178]. Thus, the efficacy of anti-angiogenic therapy may depend also on the expression of biglycan in the tumor microenvironment.

The syndecan family comprises four distinct genes encoding single-pass transmembrane protein cores. The ectodomain of syndecans is natively disordered and this characteristic allows syndecans to interact with a variety of proteins and ligands, thereby providing enrichment in their biological function [114,179]. Syndecan-1 is a key regulator of angiogenesis and this mainly occurs through the interaction with IGF-1 Receptor. This interaction is essential for the crucial cross-talk taking place between VEGFR2, the α_ν_β_3_ integrin and VE-cadherin during angiogenesis [180]. Syndecan-1 also modulates the VEGF-VEGFR2 signaling thus promoting EC proliferation and survival [181,182]. In breast cancer, stromal Syndecan-1 promotes FGF-2 and VEGF signaling and its expression in patients correlates with both vessel density and total vessel area [183]. In contrast, syndecan-2 impairs angiogenesis in human microvascular ECs [184]. Syndecans’ levels at the cell surface are regulated by proteolytic cleavage. In myeloma cells it was shown that syndecan-1 is shed and the binding of VEGF through its heparan sulfate chains stimulated tumor angiogenesis further supporting cancer growth [185]. Similar to what observed with syndecan-1, the shed ectodomains of syndecan-2 and syndecan-4 modulate angiogenesis. Shed syndecan-2 inhibits angiogenesis via a paracrine interaction with Cluster of Differentiation 148 (CD148), which in turn deactivates the α_1_β_1_ and α_2_β_1_ integrins, two main angiogenesis receptors [186]. The ectodomain of syndecan-4 is cleaved by the secreted metalloprotease A Disintegrin And Metalloproinase with Thrombospondin motifs 1 (ADAMTS1). The ectodomain in turn induces an altered distribution of actin stress fibers and a significant decrease of Ras Homolog gene family member A (RhoA) activity, thus promoting EC migration and favoring angiogenesis [187,188].

Glypicans are cell-surface PGs that share similarity with syndecans and interact with a multitude of ECM proteins, chemokines, growth factors and receptors [112]. Glypican-1 is the most prevalent member of the family expressed in ECs and in the vascular system. It is frequently overexpressed in different human malignancies including pancreatic carcinoma and breast cancer [189]. Glypican-1 is highly expressed in gliomas and this may contribute to the malignancy of this highly angiogenic tumor [190]. Glypican acts as a co-receptor or promoter of many angiogenic growth factors, including VEGF, FGFs, PDGF, heparin-binding EGF (HB-EGF), HGF, and IGF-1 [191]. A recent study indicates that the delivery of glypican-1 by nanoliposomes to the site of ischemic injury could function as an enhancer for growth factor activity, thus improving the response to local angiogenic therapies for the treatment of ischemia [192]. This represents another important example of the potential employment of ECM molecules/fragments for therapy purposes.

Lumican is localized in the peripheral blood vessels of adult human lungs and in the thickened intima of the coronary artery and displays binding affinity for α_ν_ integrins [193,194]. Lumican expression by ECs increases during the resolution phase of angiogenesis, when vessels return to a state of angiostasis [195]. The angiostatic function of this molecule is exerted through the inhibition of integrin α_2_β_1_ activity and reduction of MMP-14 expression in ECs [196]. In different tumor models lumican over-expression impaired tumor growth due to a reduced vascular density [197] likely associated with Fas-induced EC apoptosis [198].

As summarized in this section, proteoglycans are multifaceted molecules whose protein core is composed by a variety of modules. They are composed of intricate sugar chains which enable these glycoproteins to sequester and modulate the activity of different growth factors. Their GPI-anchored, transmembrane or extracellular nature enables them to affect different stages of angiogenesis during EC migration to form new vessels. Not only proteoglycans themselves offer the potential to develop new tools to halt angiogenesis, but also the enzymes that cleave these molecules, thus liberating their activity, can be viewed as a possible therapeutic target.

## 7. Hyaluronan: Not Only a Mere Glue

HA is a large polysaccharide composed of repeating *N*-acetylglucosamine and glucuronic acid disaccharide units. Under physiological conditions, HA can be composed of up to 25,000 disaccharide units, which become smaller and more dispersed in a pathological status [199]. Numerous studies have shown that HA signaling plays an important role in angiogenesis, mainly by influencing EC behavior. The biophysical functions of HA vary depending on its molecular size which is firmly regulated by the concerted activities of biosynthetic and degradation processes. For instance, low molecular weight HA (LMW-HA, generally < 200 kDa) was shown to prompt inflammation and angiogenesis [200] and stimulate EC proliferation, motility and tubule formation [201]. These oligosaccharides are able to interact with two critical EC receptors: CD44, playing a major role in EC adhesion and proliferation, and RHAMM (Receptor for HA-Mediated Motility), essential for EC invasion [202,203,204]. The role of LMW-HA in affecting angiogenesis during wound healing, diabetic mellitus and endometriosis, suggests the possibility to modulate HA as a putative therapeutic approach [205,206]. In contrast, high molecular weight HA (HMW-HA) was reported to be anti-angiogenic [207,208] and immunosuppressive [209]. Despite these evidences, their role in angiogenesis is controversial since LMW-HA was also shown to inhibit angiogenesis impairing the CXC ligand 12 (CXCL12)/CXC Receptor 4 (CXCR4) pathway, as opposed to what observed with HMW-HA [210]. In addition, HMW-HA seems to stimulate angiogenesis cooperating with versican and inducing FGF-2 expression [211]. Thus, the angiogenic properties of LMW-HA and HMW-HA may vary depending on the composition of the microenvironment. Nevertheless, a high HA expression was shown to correlate with increased angiogenesis and poor prognosis [212] and the assessment of this molecule may represent an important mean for the development of new prognostic tools [199].

Thus, given the controversial effect of this polysaccharide in angiogenesis, further preclinical analyses must be carried out to better clarify their role in blood vessel formation and verify their putative potential in clinics.

## 8. The EDEN Family: Two Members with Opposite Functions

The acronym EDEN (EMI Domain ENdowed) designates a family of ECM glycoproteins characterized by the presence of a cystein-rich EMI domain at the N-terminus [213,214,215,216].

This family of proteins can be clustered in distinct groups based on the molecular characteristics and the arrangements of the domains. The first group comprises the molecules containing the EMI domain at the N-terminus, the distinctive domain of the family, followed by a coiled-coil region and a gC1q domain at the C-terminus. This group includes MULTIMERIN1 [217], MULTIMERIN2 [218], EMILIN1 [219], and EMILIN2 [220]. The second group comprises only one molecule, EMILIN3 which lacks the gC1q domain and is required for notochord formation [221,222].

The third cluster includes two genes, Emu1 and Emu2 small collagenous molecules which share with the family only the presence of EMI domain [222].

While some of these molecules are characterized by a wide distribution in most connective tissues, like EMILIN1 and EMILIN2, the expression of other molecules such as that of MULTIMERIN2 (MMRN2) is more restricted. Despite their molecular affinity, two of them, i.e. MMRN2 and EMILIN2, exert an opposite function during angiogenesis.

MMRN2, also known as EndoGlyx-1, was identified during a screening for new antigenic markers of the vascular endothelium and was found to be expressed only at the level of the blood vessel endothelium [223]. In neoplastic tissues MMRN2 is deposited along tumor capillaries and in the “hot spots” of neoangiogenesis [223].

**Figure 2:**
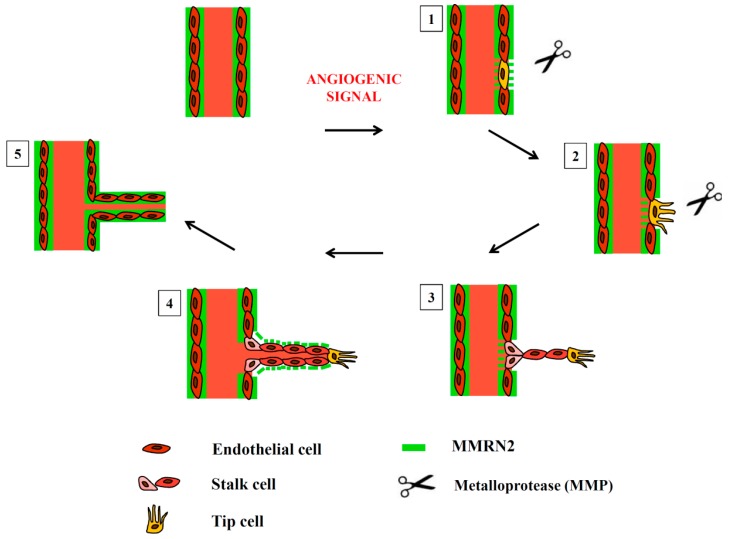
Schematic representation of the expression and degradation of MMRN2 during the steps involved in angiogenesis. (1) Following an angiogenic stimulus the basement membrane and MMRN2 are degraded by MMPs; (2) endothelial tip cells which drive sprouting angiogenesis are formed; (3) stalk cell proliferate and vessels’ lumen is formed; (4) ECs secrete MMRN2 stabilizing the vessels; (5) the quiescent state is restored.

The role of this molecule was first described in a paper by Lorenzon et al. [224], where it was found that MMRN2 primarily impinged on EC migration. This depends on its ability to sequester VEGF-A and inhibit VEGFR-2 activation [224,225]. The binding capability has been pinpointed in a region towards the N-terminus of the molecule [225]. The VEGF-binding activity primarily occurs through the carbohydrate chains, since their removal significantly compromises, the binding [225]. In addition, the molecule seems also to affect the redistribution of VEGFR2 receptor at the EC surface and to be the reservoir of different members of the VEGF family of cytokines including VEGF-C, VEGF-D and Placental Growth Factor (PlGF) [225]. When over-expressed in the tumor microenvironment the molecule and its active fragment display a potent anti-tumoral effect [224,225]. These investigations suggest that the activity is indirect and hinges on an impaired vascularization. A positive role in affecting sprouting angiogenesis has also been reported, thanks to the interaction with the tumor endothelial marker CLEC14A [226,227]. This indicates that the molecule may display different effects during angiogenesis, depending on the stage of vessels development. Anyhow, given the presence of the molecule along all the blood vessels it is conceivable that it might exert a homeostatic role, thus halting the development of new vessels unless a strong pro-angiogenic signal is released. Under these conditions, the protein could be degraded by the proteases secreted by ECs during migration to allow an efficient sprouting. Preliminary results generated in our laboratory support this hypothesis, still we have only begun to scratch the surface to assess the role of this molecule in angiogenesis and vessel homeostasis (Figure 2).

EMILIN2 was cloned following a two-yeast hybrid screening and is characterized by a proline-rich segment between the coiled-coil region and the collagenous stalk [220]. EMILIN2 displays a low expression in adult aorta, small intestine and appendix, whereas the highest levels of the protein can be detected in fetal heart starting at E8.5 and reaching the highest levels at E11.5 and adult lung mice [228]. At the functional level, EMILIN2 significantly impairs the growth of a number of tumor types inducing tumor cell apoptotic death and impairing Wnt signaling [11,15]. The *Emilin2* gene is frequently methylated in breast, lung and colorectal tumors and this suppression correlates with poor clinical outcome and increased limph node metastasis in breast cancer patients [229].

**Figure 3:**
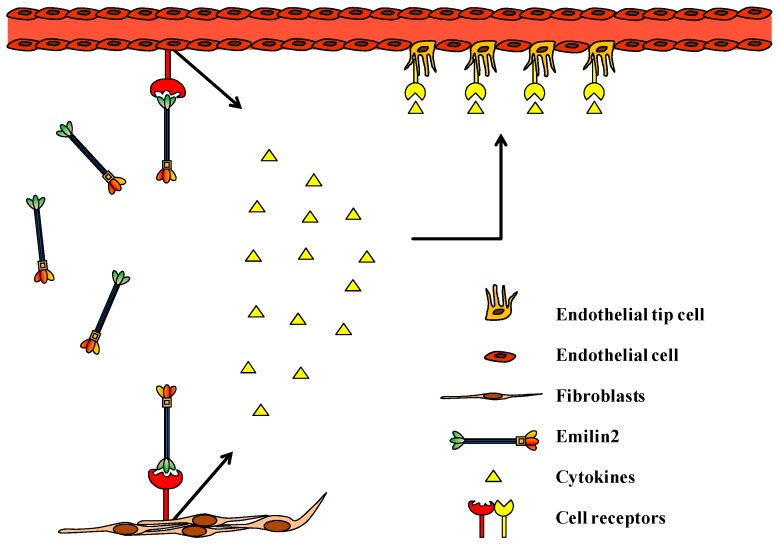
EMILIN2 stimulates angiogenesis via an RTK-dependent cytokine production. Schematic representation of the molecular mechanisms elicited by EMILIN2. The molecule interacts directly with membrane receptors present in both ECs and fibroblast. This leads to the activation of an intracellular signaling cascade that results in the overproduction of angiogenic cytokines that, in turn, boost EC proliferation and migration.

Interestingly, and unexpectedly, EMILIN-2 also stimulates the development of new vessels [230,231]. The molecular mechanisms by which EMILIN2 affect ECs behavior are being investigated in our laboratory and involve the over-production of cytokines which, in turn, promote EC proliferation and migration (Figure 3). Furthermore, tumor vessels developed in EMILIN2-deprived microenvironments display a worsen integrity of the basal lamina suggesting that it might also affect vessel perfusion and drug delivery (unpublished observations).

Thus, despite the similar domain arrangements, EMILIN2 and MMRN2 exert opposite functions. This may depend on the fact that the angiogenic activity of MMRN2 occurs through the coiled-coil region, which shares low homology with that of EMILIN2, of which the pro-angiogenic region has not been identified yet.

## 9. The CCN Family of Proteins as Regulators of Vascular Development and Pathological Angiogenesis

The Connective tissue growth factor Cystein rich protein and Nephroblastoma overexpressed gene (CCN) family of proteins includes six members (CCN1–CCN6) that share conserved functional domains. These proteins have been shown to be reservoirs of growth factors and to promote intracellular signaling. This occurs through the interaction with cell surface integrins, receptors, or other ECM molecules [232]. These molecules have been extensively studied and their role in the modulation of the proliferation, migration and adhesion of EC, among other cells, established. Despite the homology, the function of the different CCN proteins is exclusive due to the specific expression patterns [233]. For this reason, their correct deposition in many physiologic processes is essential and an unbalanced secretion of these molecules often leads to severe disorders contributing to cancer progression and the onset of vascular diseases.

The CCN1 (CYR61) protein is expressed by ECs and vascular smooth muscle cells (VMSC); cardiac expression occurs at E8.5 and persists until E11.5 during mouse embryo development. The importance of this molecule in angiogenesis is highlighted by the fact that CCN1 null mice die at E14.5 due to vascular defects [234]. The mechanism of action involves the engagement of integrin α_v_β_3_ and the promotion of EC adhesion and migration [235]. CCN1 stimulates tumor growth and is associated with an increased intra-tumor vascularization [235]. By promoting the differentiation of progenitor ECs, CCN1 supports the re-endothelialization after vascular injury [236]. In addition, by targeting VEGF, Src homology 2 domain phosphatase-1 and Notch signaling, CCN1 affects the development of retinal vessels [237].

The CCN2 protein (CTGF) shares with CCN1 similar expression patterns and it is not only expressed by ECs and VSMC but also by pericytes and regulates the interaction of these cells with ECs [238]. CCN2 null mice die shortly after birth due to severe skeletal and vascular defects associated with an impaired pericyte recruitment and basement membrane organization [239]. Given the importance of pericytes in maintaining vascular stability and affecting their efficiency, it is likely that an altered expression of the molecule may also affect drug delivery. CCN2 induces a HIF-1α-dependent VEGF expression and this occurs through the up-regulation of miR-210 via the activation of Phosphoinositol 3 kinase (PI3K), PKB, extracellular signal-regulated kinase (ERK) and nuclear factor kappa-light-chain-enhancer of activated B cells (NF-κB)/ETS-domain containing protein 1 (ELK1), leading to increased angiogenesis [240].

The CCN3 (NOV) protein is structurally similar to CCN1 and 2 but displays a divergent function protecting from aberrant excessive vessel growth [241]. CCN3 supports EC adhesion and/or migration through integrins α_ν_β_3_, α_5_β_1_, α_6_β_1_ and through heparan sulfate proteoglycans inducing corneal vascularization [242]. It was recently demonstrated that CCN3 plays also a key role in the development of abdominal aortic aneurysm [243]. CCN3 stimulates the expression of VEGF in prostate cancer cells activating the focal adhesion kinase (FAK)/PKB/NF-κB signaling pathway. Furthermore, CCN3 stimulates the recruitment of macrophages which are skewed to an M2 phenotype, thus leading to enhanced tumor angiogenesis [244]. By modifying this key cellular microenvironmental component CCN3 is thus able to deeply affect cancer growth both directly, affecting cancer cell behavior, and indirectly, affecting angiogenesis.

The CCN4 (WISP1) protein is a major regulator of skeletal development and only recently a role in VSMC migration and proliferation has been recognized [245]. A stimulation of VEGF-A expression was also demonstrated for CCN4 and it occurs via the engagement of integrin α_v_β_3_ and the consequent activation of the FAK/c-Src pathway. This leads to the transactivation of the EGFR/ERK/HIF1-α, which prompts the development of new blood vessels [246].

The CCN5 (WISP2) protein is expressed early and persists throughout embryonic development; it is expressed by EC, VSMC and heart myocardium suggesting a role in vascular function [247]. Indeed, recent evidences indicate that CCN5 exerts potent anti-angiogenic effects in an aortic ring vessel outgrowth model, and this angiostatic activity is abrogated by its cleavage [248]. Thus, its cleavage may be a mechanism through which, under a strong angiogenic stimulus, the potent anti-angiogenic properties of CCN5 are turned off.

Unlike all the other members of the family, CCN6 (WISP3) has not so far been demonstrated to play a role in vascular development.

Also for this family of molecules the evidences indicating an involvement in angiogenesis are growing. Given their dual action on cancer cells and vascular cells they thus represent a promising source for the development of new anti-tumor drugs. As indicated above CCN2, CCN3 and CCN4 induce VEGF activation. Thus, in case of high expression levels of these molecules, it should be taken into account that anti-angiogenic therapies aimed at blocking VEGF activity may be hampered.

## 10. Conclusions

In this review, we report same examples of the intricate and complex regulations exerted by components of the ECM and deeply affecting the angiogenic process. For this reason, one can conceive that the concentration and processing of these molecules in the tumor microenvironment could be taken into account as an approach to predict the therapeutic efficacy. In fact, through their action these molecules can impinge on drug delivery and efficacy by modifying the vessels’ efficiency. In addition, for their prominent role in vessel formation the ECM composition should be taken into account to predict the efficacy of anti-angiogenic approaches. Finally, their endogenous nature offers the potential for the development of new more efficacious and less toxic treatments of angiogenesis-dependent diseases.

## Appendix A

**Table A1:** List reporting the main ECM molecules involved in angiogenesis.

ECM Proteins	Fragments	Anti-Angiogenic Activity	Pro-Angiogenic Activity	References
Trombospondins				
TSP-1		√		[27,28,30,32,33,34,35,36,37,38,39,40,41,42,43,44]
TSP-2		√		[29,31,44]
Fibronectin				
Fibronectin			√	[51,52,53,54,55,56]
Collagens				
Type I			√	[58,59,60,61]
Type IV			√	[67]
	arresten	√		[65,71]
	canstatin	√		[72,73,74,75]
	tumstatin	√		[76,77]
Type XV	restin	√		[80,81,82]
Type XVIII	endostatin	√		[81,83,84,85,86,87,92]
Laminins				
Laminin 411 and 421			√	[101,102,103,105]
Laminin 511		√		[104,106]
Proteoglicans				
Perlecan			√	[112,133,134,135,136,137,138,139,140,141,142]
	endorepellin	√		[148,149,150,151,152,153,154,155,156,157]
Decorin		√		[162,163,164,165,166,167,168,169,170,171,172,173,174,175]
			√	[159,160,161,163,164,165,169,170,171,172,173,174,175]
Biglycan			√	[176,177,178]
Syndecan 1			√	[181,182,183,185]
Syndecan 2		√		[184,186]
Syndecan 4			√	[187,188]
Glypicans			√	[189,190,191,192]
Lumican		√		[196,197,198]
Hyaluronan				
LMW-HA			√	[200,201,202,203,204,205,206]
		√		[210]
HMW-HA		√		[207,208]
			√	[211]
EDEN Family				
Multimerin 2		√		[224,225]
			√	[226,227]
	Δ2 fragment	√		[225]
Emilin 2			√	[230,231]
CCN Family				
CCN1			√	[235,236,237]
CCN2			√	[239,240]
CCN3			√	[241,242,243,244]
CCN4			√	[245,246]
CCN5		√		[248]

ECM: Extracellular matrix; TSP: Thrombospondin; LMW-HA: Low Molecular Weight Hyaluronan; HMW-HA: High Molecular Weight Hyaluronan; EDEN: EMI Domain ENdowed; CCN: Connective tissue growth factor Cystein rich protein and Nephroblastoma overexpressed gene.
